# Research progress of exosomes in renal ischemia-reperfusion injury

**DOI:** 10.3389/fphar.2026.1763241

**Published:** 2026-05-12

**Authors:** Supeng Tai, Weibo Wang, Guangyue Luo, Junyue Tao, Chaozhao Liang, Jun Zhou

**Affiliations:** 1 Department of Urology, The First Affiliated Hospital of Anhui Medical University, Hefei, Anhui, China; 2 Institute of Urology, Anhui Medical University, Hefei, Anhui, China; 3 Anhui Province Key Laboratory of Genitourinary Diseases, Anhui Medical University, Hefei, Anhui, China

**Keywords:** drug delivery, exosome, hydrogels, programmed cell death, renal ischemia-reperfusion injury

## Abstract

Renal ischemia-reperfusion injury (RIRI), which causes renal damage that occurs when blood flow is restored after a period of reduced perfusion, is a common cause of acute kidney injury. As the disease progresses, treatment options become increasingly limited, highlighting the urgent need for new therapeutic approaches. Among these, exosomes (Exos) have shown great potential in the prevention and treatment of this condition. Exos are nano-sized vesicles of endosomal origin, typically less than 200 nm in diameter, and have emerged as key mediators in diverse pathophysiological processes. This article provides a comprehensive narrative review of the mechanisms of various types of Exos in renal ischemia-reperfusion injury and the advancements in exosome therapy, and details both their renoprotective mechanisms and potential pathogenic effects. Furthermore, we highlight advanced bioengineering strategies, delivery platforms, and diagnostic potential. By explicitly addressing translational barriers and the need for rigorous methodological standardization, we aim to provide comprehensive insights for advancing exosome-based RIRI management from bench to clinic.

## 1 Introduction

**FIGURE 1:**
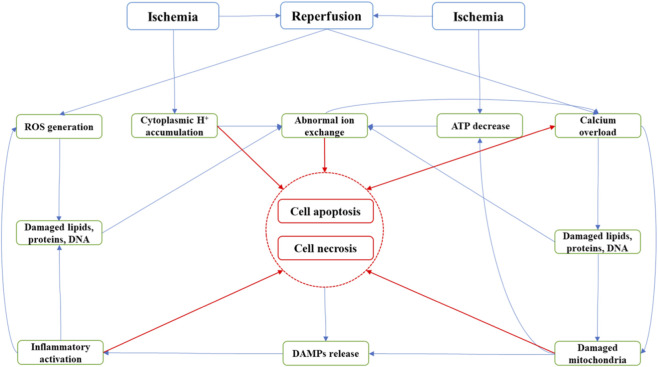
The illustration of the occurrence process of ischemia-reperfusion and the connection between key pathophysiological mechanisms (Note: This figure is from our previously published work. Details are as follows: Wang W. et al. (2024).

Renal ischemia–reperfusion injury (RIRI) is renal damage that occurs when blood flow is restored after a period of reduced perfusion. After ischemic injury, the abrupt reintroduction of oxygen paradoxically exacerbates cellular dysfunction through mechanisms including uncontrolled propagation of oxidative stress, dysregulated inflammatory cascades, and activation of programmed cell death pathways, ultimately leading to secondary tissue injury and AKI (Wang W. et al., 2024). Therefore, RIRI is a major cause of acute kidney injury (AKI) and is frequently encountered in kidney transplantation, partial nephrectomy and diverse forms of shock. A proportion of patients with AKI progress to chronic kidney dysfunction and ultimately end-stage renal disease (ESRD), which is largely irreversible. Currently, a limited understanding of the pathogenesis of RIRI and the absence of well-defined therapeutic targets restrict the efficacy of conventional treatments (Si et al., 2026). Clinical management therefore remains largely conservative and supportive. Once the disease advances to ESRD, patients have no option but dialysis or kidney transplantation, options that are associated with a poor prognosis. The pathophysiology of RIRI is complex and involves mitochondrial dysfunction, calcium overload, necroptosis, pyroptosis and injury to tubular and endothelial cells (Salvadori et al., 2015). During IRI, the reintroduction of oxygen triggers a burst of reactive oxygen species (ROS) from sources like mitochondria and xanthine oxidase, which damage lipids, proteins, and DNA. This oxidative injury disrupts membranes and ion pumps, leading to calcium overload as damaged cells fail to expel Ca2+ and instead exchange accumulated intracellular Na + for extracellular Ca2+ upon reperfusion. The resulting calcium surge activates degradative enzymes and opens the mitochondrial permeability transition pore (mPTP), while mitochondrial ROS from succinate-driven reverse electron transport (RET) further amplifies damage. Concurrently, damaged cells release DAMPs that trigger sterile inflammation, recruiting neutrophils that release additional ROS and proteases, which can cause microvascular obstruction and the “no-reflow” phenomenon, thereby establishing a vicious cycle of injury (Wang W. et al., 2024) (Figure 1). Given the multifaceted pathophysiology of RIRI, novel therapeutic strategies are urgently needed. In this context, Exos have emerged as promising candidates.

Exos are 30–200 nm extracellular vesicles derived from the endosomal pathway, where intraluminal vesicles bud inward to form multivesicular bodies (MVBs) that subsequently fuse with the plasma membrane to release their cargo. Ubiquitously distributed in biofluids, these nanoscale vesicles carry diverse molecular cargos, including regulatory proteins, coding/non-coding RNAs, and bioactive lipids. By facilitating intercellular communication and material exchange, Exos exert dual regulatory effects on tissue homeostasis and disease progression (Wang W. et al., 2024). Once regarded as inert cellular debris, Exos are now recognized as active participants in fundamental biological processes. Released by a broad range of cell types, they selectively package and secrete specific cargoes to modulate distinct biological functions. Among these cell types, stem cells show particularly robust Exos secretion and display small size, low immunogenicity, prolonged circulation time and favorable storage stability. Exos derived from stem cells exert pivotal regulatory effects in RIRI (Hade et al., 2021). More recently, Exos derived from non-stem cell populations have also been shown to preferentially target renal tissue and to modulate both RIRI and subsequent renal fibrosis. Together, these observations highlight the considerable therapeutic potential of Exos in RIRI and support their development as a novel strategy for the future management of this condition. Reactive oxygen species (ROS) serve as key molecular mediators linking oxidative damage to immune activation, thereby establishing a positive feedback loop of “oxidative stress → inflammation → further oxidative stress” (Liu et al., 2026). Excessive ROS damage cellular components (e.g., lipids, DNA), activate inflammatory signaling pathways such as NF-κB, and induce the release of pro-inflammatory cytokines. Conversely, activated immune cells (e.g., macrophages) generate additional ROS, which further aggravate oxidative injury (Bungau et al., 2023). Therefore, targeting the crosstalk nodes among these three processes—such as RIPK1, mitochondrial ROS, and GSDM proteins—can synergistically suppress oxidative damage, inflammatory storms, and pathological cell death.

Although the focus of this review is RIRI, a substantial fraction of mechanistic evidence relevant to kidney injury biology comes from non-ischemic models, including sepsis-associated AKI, diabetic kidney disease, and cisplatin-induced AKI. We include these studies when they interrogate pathways that are plausibly shared with RIRI and when they generate hypotheses that can be tested directly in ischemia-reperfusion systems. Where evidence derives from non-RIRI contexts, we identify the model explicitly and interpret the findings as supportive rather than RIRI-specific. For example, one study of exosome-mediated protection in sepsis-associated AKI noted that its observations might inform RIRI research (Gao et al., 2020). Work in cisplatin-induced AKI has also been used to probe pyroptosis in non-ischemic injury and provides mechanistic leads that warrant validation in RIRI (Zhu et al., 2023). The in-depth exploration of non-ischemic models not only reveals shared molecular targets but also highlights translatable intervention strategies (such as Exos engineering and combination therapies), thereby providing direct experimental evidence and theoretical support for deepening the mechanistic understanding and therapeutic development of RIRI.

## 2 Cellular sources and classification of Exos in RIRI

Stem cell-derived Exos, particularly those from mesenchymal stem cells (MSCs), are pivotal extracellular vesicles in the regulation of RIRI progression. Recent evidence indicates that Exos from urine-derived stem cells (USCs) and renal tubular epithelial cells (TECs) contribute to the onset, progression and therapeutic modulation of RIRI. MSCs exhibit robust self-renewal, multipotent differentiation, high proliferative capacity and strong immunomodulatory properties.

MSCs are mainly isolated from bone marrow, adipose tissue and umbilical cord, and despite their distinct origins these populations share similar differentiation capacity and biological functions. However, abnormalities in telomere regulation and dysregulated expression of cell cycle–related genes may predispose stem cells to chromosomal instability and increase the risk of tumorigenesis. MSC-derived Exos (MSC-Exos) carry abundant proteins, RNAs, anti-inflammatory mediators and proangiogenic factors, and display tissue-homing capacity, low immunogenicity, high stability and controllable cargo release, which supports their broad use in biomedical research and therapy (Baglio et al., 2012). Common MSC subtypes include bone marrow-derived MSCs (BMSCs), adipose-derived MSCs (ADMSCs) and umbilical cord-derived MSCs (UCMSCs). Additional stem cell populations include kidney-derived MSCs (KMSCs) and human Wharton’s jelly-derived MSCs (hWJMSCs).

USCs can be isolated noninvasively from urine and arise mainly from renal podocytes or parietal epithelial cells. They show strong potential for renal repair and regeneration and readily differentiate into kidney-associated lineages.

Exos originating from epithelial and endothelial tissues have also been widely investigated, particularly those derived from renal TECs, human amniotic epithelial cells (hAECs) and vascular endothelial cells. TEC-derived Exos (TEC-Exos) are released directly from renal TECs after RIRI. hAEC-derived Exos (hAEC-Exos), which arise from the inner amniotic layer formed by the early blastocyst inner cell mass, display potent regenerative and cytoprotective properties. Endothelial cell-derived Exos, including those from human umbilical vein endothelial cells (hUVECs) and endothelial progenitor cells (EPCs), play essential roles in vascular homeostasis and pathological vascular remodelling.

## 3 Mechanistic actions of Exos in RIRI

The renoprotective actions of Exos in RIRI are mediated mainly by attenuation of oxidative stress, suppression of inflammatory responses, inhibition of apoptosis and the promotion of angiogenesis and renal TEC proliferation (Table 1).

**TABLE 1:** Comprehensive overview of Exos derived from diverse cellular sources and their therapeutic mechanisms in renal injury, including anti-inflammatory, anti-oxidative, anti-apoptotic, pro-angiogenic, anti-fibrotic, and immunomodulatory effects.

Source cell phenotype (effect)	Target/Key	Molecules or pathways
Human umbilical cord MSCs (hHCMSC)	Antioxidant	Downregulate NOX2, suppress ROS generation
Human umbilical cord MSCs (hHCMSC)	Anti-apoptosis	miR-100-5p activates FKBP5/AKT signaling miR-125b-5p downregulates p53
Human umbilical cord MSCs (hHCMSC)	Inhibit pyroptosis	Downregulate GSDMD, caspase-1, NLRP3, IL-1β Activate TRPC6/PARP1 pathway,miR-874-3p targets RIPK1
Human umbilical cord MSCs (hHCMSC)	Promote angiogenesis & cell proliferation	Upregulate klotho, BMP7, VEGFA
Human Wharton’s jelly MSCs (hWJMSC)	Antioxidant & anti-apoptosis	miR-30 inhibits mitochondrial fission
Bone marrow MSCs (BMSC)	Anti-inflammatory	miR-223-3p targets NLRP3, promotes mitophagy
Bone marrow MSCs (BMSC)	Anti-apoptosis	miR-125b-5p downregulates p53
Bone marrow MSCs (BMSC) engineered	Anti-inflammatory	Overexpress indoleamine 2,3-dioxygenase (Ido)
Adipose-derived MSCs (ADMSC)	Anti-inflammatory & anti-apoptosis	miR-342-5p targets TLR9, enhances autophagy, Activate SIRT1, inhibit NF-κB signaling
Umbilical cord MSCs (UCMSC)	Anti-fibrotic	Inhibit wnt/β-catenin signaling
Urine-derived stem cells (USC)	Anti-inflammatory & anti-fibrotic	miR-146a-5p targets IRAK1 Inhibits NF-κB circRNA ATG7 sponges miR-4500, releases SOCS1 to inhibit STAT3
Urine-derived stem cells (USC)	Anti-fibrotic & promote proliferation	miR-122-5p targets SOX2, activates PI3K/AKT and MAPK/ERK
Urine-derived stem cells (USC)	Anti-apoptosis	miR-216a-5p targets PTEN, activates AKT
Urine-derived stem cells (USC)	Promote cell proliferation	Activates circDENND4C/miR-138-5p/FOXO3a axis
Urine-derived stem cells (USC)	Inhibit ferroptosis	lncRNA TUG1 interacts with SRSF1, degrades ACSL4 mRNA
Renal tubular epithelial cells (TEC)	Antioxidant & anti-apoptosis	miR-146a-5p and miR-200a-3p decrease ROS, enhance SOD/CAT,miR-20a-5p suppresses oxidative stress and apoptosis
Renal tubular epithelial cells (TEC)	Pro-inflammatory	miR-93-3p (*via* CREB1/CRTC2) suppresses NFAT5 Activates NIK/NF-κb2 Activates miR-106b-5p/ATL3 signaling promotes M1 polarization
Renal tubular epithelial cells (TEC)	Promote proliferation & anti-inflammatory	Overexpress CD26, downregulate CXCR4/SDF1 axis
Human amniotic epithelial cells (hAEC)	Anti-inflammatory & promote M2 polarization	Increase IL-4, IL-13; decrease TNF-α, IFN-γ
Human amniotic epithelial cells (hAEC)	Anti-inflammatory	Activate ERK1/2 pathway
Human umbilical vein endothelial cells (hUVEC)	Anti-inflammatory	Suppress MMP-2 and MMP-9 expression
Fibroblastic reticular cells	Inhibit pyroptosis & promote mitophagy	Enriched in CD5L, enhances PINK1-PARKIN mitophagy, inhibits NLRP3
Endometrial regenerative cells	Anti-inflammatory & immunomodulatory	Express CD73, hydrolyzes ATP to adenosine, activates MAPK pathway
Parietal epithelial cells (PEC)	Promote cell cycle progression & anti-fibrotic	Let-7b-5p targets TGFβR1 and ARID3a, downregulates p21 and p27

### 3.1 Antioxidant responses

During RIRI progression, abrupt reintroduction of oxygen triggers excessive generation of reactive oxygen species (ROS), including superoxide anion (O^2−^), hydrogen peroxide (H_2_O_2_) and hydroxyl radicals (•OH) (Schieber and Chandel, 2014). These ROS attack membrane lipids, proteins and DNA, causing structural and functional injury, disruption of membrane integrity, leakage of intracellular contents and ultimately cell death. DuringIRI, ROS are generated mainly through mitochondrial electron transport, xanthine oxidase activity in endothelial cells, leukocyte NADPH/NADH oxidase systems, monoamine oxidase–mediated catecholamine autoxidation and activation of inducible nitric oxide synthase (iNOS) (Kloner et al., 1989; Zhao et al., 2010; Granger and Kvietys, 2015; Pantic et al., 2021). Because of their high reactivity, ROS readily interact with diverse cellular constituents and induce metabolic disturbance. Membrane phospholipids enriched in polyunsaturated fatty acids are especially vulnerable to ROS-mediated peroxidation, which leads to loss of cellular and organelle membrane integrity, leakage of intracellular contents and cell death (Siems et al., 1995). Furthermore, ROS oxidize sulfhydryl groups on polypeptide chains, leading to protein misfolding, denaturation, aggregation, degradation and peptide backbone cleavage, ultimately impairing the function of enzymes, receptors and ion chan^nels^ (Stadtman and Levine, 2003). ROS also induce oxidative base modification, DNA strand breaks and cross-link formation, thereby damaging both nuclear and mitochondrial DNA. Consequently, the antioxidant properties of Exos can attenuate IRI-induced oxidative damage and facilitate renal tissue repair.

**FIGURE 2:**
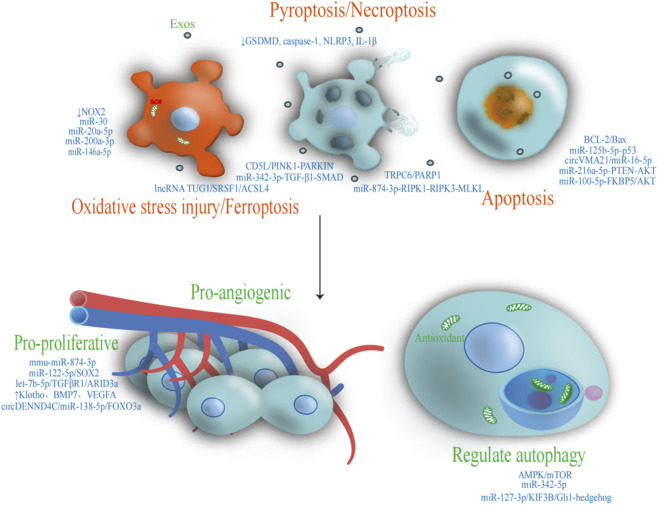
Schematic representation of the multifaceted therapeutic effects of Exos: inhibition of pyroptosis, necroptosis, and apoptosis; attenuation of oxidative stress and ferroptosis; and modulation of autophagy to enhance angiogenesis and cell proliferation.

In preclinical studies, Zhang et al. showed that Exos derived from human umbilical cord MSCs (hHCMSC-Exos) attenuate oxidative stress-induced injury and promote renal functional recovery by downregulating NADPH oxidase 2 (NOX2) and suppressing ROS generation (Zhang et al., 2014). Gu et al. reported that Exos derived from hWJMSCs (hWJMSC-Exos) deliver miR-30 to inhibit mitochondrial fission, reduce intracellular oxidative stress, limit the release of pro-apoptotic factors, confer renoprotection and slow the progression of RIRI (Gu et al., 2016). In models of calcium oxalate crystal-induced renal injury, exosomal miRNAs derived from normal renal TECs, such as miR-146a-5p and miR-200a-3p, mitigate oxidative stress by decreasing ROS and malondialdehyde (MDA) and enhancing superoxide dismutase (SOD) and catalase (CAT) activity, ultimately conferring renoprotective effects (Yang Y. et al., 2025). Consistently, Yu et al. showed that miR-20a-5p enriched in TEC-Exos suppresses oxidative stress and apoptosis, thereby protecting against acute tubular injury (Yu et al., 2020) (Figure 2).

### 3.2 Anti-inflammatory responses

RIRI triggers a cascade of inflammatory responses that broadly activate both innate and adaptive immunity. This sterile inflammation is independent of pathogen invasion and is driven by ischemia-induced pro-inflammatory signaling cascades (Chen and Nuñez, 2010). Several key mechanisms are involved. First, damage-associated molecular patterns (DAMPs) engage pattern recognition receptors (PRRs), which activates the complement cascade, upregulates adhesion molecules and promotes cytokine release (Ioannou et al., 2011; Tang et al., 2022). Second, neutrophils and other leukocytes roll and adhere to activated endothelium and then transmigrate into ischemic and inflamed tissue (Nourshargh and Alon, 2014). Third, neutrophil accumulation, endothelial swelling, platelet adhesion and microthrombus formation contribute to the no-reflow phenomenon, causing microvascular obstruction, tissue oedema and worsening hypoxia (Zhang et al., 2024). Finally, activated leukocytes and injured endothelial cells release ROS and proteases that damage surrounding tissue, while DAMPs released from dying cells further recruit leukocytes and activate both innate and adaptive immunity, sustaining a vicious cycle of inflammation and tissue injury (Iyer et al., 2009).

Growing evidence indicates that Exos modulate these inflammatory processes. Xie et al. reported that BMSC-Exos shift macrophage polarization from a pro-inflammatory M1 phenotype toward an anti-inflammatory M2 phenotype and enhance the production of anti-inflammatory mediators (Xie et al., 2022). Sun et al. showed that BMSC-derived miR-223-3p directly targets NLRP3, promotes mitophagy and reduces inflammasome-mediated inflammatory responses (Sun et al., 2022a). Li et al. showed that USCs ameliorate RIRI in rats, and mechanistic studies revealed that miR-146a-5p enriched in USC-derived Exos (USC-Exos) targets the 3′UTR of IRAK1, suppresses downstream NF-κB activation and attenuates inflammatory cell infiltration (Li et al., 2020). Ren et al. showed that intravenous administration of hAEC-Exos increases IL-4 and IL-13, reduces TNF-α and IFN-γ and promotes macrophage polarization from an M1 to an M2 phenotype, which alleviates tubular apoptosis and necrosis and enhances regenerative proliferation (Ren et al., 2020). hAEC-Exos also activate the ERK1/2 pathway to further ameliorate RIRI (Lim et al., 2022). Zhao et al. observed that Exos derived from hUVECs (hUVEC-Exos) attenuate RIRI by suppressing MMP-2 and MMP-9 expression (Zhao, 2022).

**FIGURE 3:**
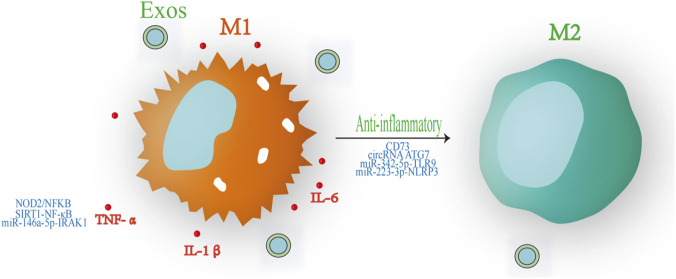
Schematic illustration of the anti-inflammatory mechanisms of Exos, including the inhibition of pro-inflammatory cytokines (TNF-α, IL-6, and IL-1β) and the induction of M2 macrophage polarization.

Additional work has shown that USC-Exos carry circRNA ATG7, which acts as a molecular sponge for miR-4500, releasing SOCS1 from miRNA-mediated repression and subsequently inhibiting STAT3 signaling. This mechanism promotes macrophage polarization toward an M2 phenotype and attenuates the progression of diabetic nephropathy (Sun et al., 2024). In sepsis-associated AKI, Exos released by ADMSCs (ADMSC-Exos) deliver miR-342-5p, which targets and suppresses Toll-like receptor 9 (TLR9), enhances autophagy and attenuates inflammatory responses and renal damage (Liu W. et al., 2023). Exos derived from endometrial regenerative cells express CD73 on their surface and hydrolyze pro-inflammatory ATP into immunosuppressive adenosine. By activating the mitogen-activated protein kinase (MAPK) pathway, these Exos promote macrophage polarization from an M1 to an M2 phenotype and modulate T cell responses, ultimately attenuating renal inflammation, tissue injury and functional impairment (Shao et al., 2025). Furthermore, MSC-Exos suppress the NOD2/NF-κB signaling axis, which reduces podocyte inflammation, oxidative stress and apoptosis in high-glucose and diabetic nephropathy models (Wang Y. et al., 2024). In sepsis-induced AKI, ADMSC-Exos activate SIRT1 and inhibit NF-κB signaling, conferring anti-inflammatory and anti-apoptotic effects and improving renal microcirculation. These findings provide a valuable reference for future research on similar pathways in RIRI (Gao et al., 2020)^.^ Exos from hypoxia-preconditioned adipose MSCs that carry circRNA mmu_circ_0001295 further attenuate renal vascular leakage, preserve kidney function and mitigate systemic inflammation in septic mouse models (Cao et al., 2022) (Figure 3).

### 3.3 Programmed cell death

Apoptosis, a non-inflammatory form of programmed cell death, is a key mechanism in the onset and progression of RIRI. Wang et al. reported that BMSCs suppress apoptosis of renal TECs, reduce pro-inflammatory cytokine secretion and increase expression of the autophagy marker LC3B and the autophagy-related proteins ATG5 and ATG7 in rat renal epithelial cells (Wang et al., 2017). Chen et al. showed that miR-100-5p delivered by hHCMSC-Exos activates the FKBP5/AKT signaling pathway and inhibits apoptosis in HK-2 cells (Chen et al., 2024). Cao et al. showed that hHCMSC-Exos-derived miR-125b-5p suppresses p53 in TECs, upregulates the cell cycle regulators CDK1 and cyclin B1 and shifts the balance between anti-apoptotic BCL-2 and pro-apoptotic Bax toward survival, thereby reducing TEC apoptosis (Cao et al., 2021). Zhang et al. reported that miR-216a-5p enriched in USC-Exos targets PTEN, activates AKT signaling and suppresses apoptosis of renal TECs (Zhang et al., 2020). Additionally, Exos derived from adipose MSCs (ADMSC-Exos) that deliver circVMA21, which sponges miR-16-5p attenuate LPS-induced apoptosis, inflammation and aerobic glycolysis in HK-2 cells and mitigate sepsis-associated AKI (He et al., 2023). MSC-Exos delivering miR-186-5p target Smad5, inhibit TGF-β1/Smad5 signaling, limit extracellular matrix deposition and epithelial–mesenchymal transition, reduce apoptosis and attenuate renal fibrosis (Yang et al., 2022). Circulating Exos from rats with myocardial infarction that carry miR-1-3p target ATG13 and activate AKT signaling, thereby suppressing both apoptosis and excessive autophagy in renal TECs (Zhao et al., 2021). Exos derived from curcumin-preconditioned BMSCs, which are enriched in the m6A demethylase FTO, reduce m6A methylation of the OXSR1 gene and attenuate apoptosis, inflammation and oxidative stress in sepsis-associated AKI (Yang T. et al., 2025).

Exos are also key modulators of pyroptosis and necroptosis. Wan et al. showed that hHCMSC-Exos suppress pyroptosis in rat kidneys subjected to RIRI by downregulating GSDMD, caspase-1, NLRP3 and IL-1β (Wan et al., 2023). Exos originating from fibroblastic reticular cells, which are enriched in CD5L, enhance PINK1-PARKIN-mediated mitophagy and inhibit NLRP3 inflammasome activation, thereby mitigating pyroptosis and renal injury (Li et al., 2024). Umbilical cord MSC-Exos delivering miR-342-3p inhibit TGF-β1/SMAD signaling, attenuate pyroptosis in TECs and slow the progression of chronic kidney failure (Yang J. et al., 2025). Guo et al. reported that hHCMSC-derived Exos activate the TRPC6/PARP1 pathway and reduce necroptosis induced by RIRI in rats (Guo, 2023). Other work has shown that hHCMSC-Exos deliver miR-874-3p, which targets RIPK1 to inhibit the RIPK1/RIPK3/MLKL necroptosis pathway and downstream PGAM5/Drp1-mediated mitochondrial fission, thereby limiting tubular epithelial injury and promoting repair (Yu et al., 2023).

In addition, Exos have emerged as important regulators of ferroptosis, an iron-dependent form of programmed cell death. Ferroptosis is driven by iron-dependent lipid peroxidation and is increasingly recognized as a critical contributor to RIRI. Sun et al. reported that human USC-Exos (hUSC-Exos) are highly enriched in the long non-coding RNA TUG1. Once delivered to renal TECs, TUG1 interacts with the RNA-binding protein SRSF1 and promotes degradation of ACSL4 mRNA, a key driver of ferroptosis. This suppresses ACSL4-mediated ferroptosis and results in marked attenuation of RIRI in both *in vivo* and *in vitro* models (Sun et al., 2022b) (Figure 2).

### 3.4 Angiogenesis and cell proliferation

Exos modulate angiogenesis and cell proliferation in the context of RIRI. Huang et al. reported that hHCMSC-Exos upregulate Klotho, bone morphogenetic protein 7 (BMP7), and vascular endothelial growth factor A (VEGFA) and its receptor in mice with RIRI, thereby enhancing endothelial and TEC proliferation, suppressing pro-inflammatory cytokine expression, and leading to a marked improvement in renal function (Huang et al., 2022). Moreover, USC-Exos have been shown to activate the circDENND4C/miR-138-5p/FOXO3a axis to promote cell proliferation, while concomitantly inhibiting NLRP3 inflammasome activation and apoptosis, thereby significantly improving outcomes in RIRI (Yang et al., 2024).

A pioneering study demonstrated that Exos derived from parietal epithelial cells (PEC-Exos), enriched in let-7b-5p, target and repress TGFβR1 and ARID3a, leading to downregulation of the cell cycle inhibitors p21 and p27. This allows G1-arrested TECs to re-enter the cell cycle (G1/S transition), thereby promoting proliferation and self-repair, improving renal function, reducing tubulointerstitial collagen deposition and fibrosis, and attenuating inflammatory responses (Song et al., 2024). In models of partial bladder outlet obstruction–induced renal injury, UCMSC-Exos preferentially accumulate in damaged renal tissue, where they inhibit Wnt/β-catenin signaling, suppress pathological proliferation and the expression of injury and fibrosis markers such as α-SMA and PCNA, and ameliorate renal fibrotic remodeling (Wang Z. et al., 2023). In cisplatin-induced AKI models, bone marrow MSC-derived Exos deliver mmu-miR-874-3p, which targets and suppresses FZD5, leading to activation of Wnt/β-catenin signaling, enhanced angiogenesis, and attenuation of renal injury (Kong et al., 2025). Furthermore, USC-derived Exos have been shown to deliver miR-122-5p, which directly targets the 3′UTR of SOX2, thereby suppressing its expression, activating PI3K/AKT and MAPK/ERK signaling, and restraining excessive p38 MAPK activation. Collectively, these effects confer robust anti-fibrotic and anti-inflammatory activity and promote cell proliferation and angiogenesis in both *in vitro* and *in vivo* models (Lu et al., 2024) (Figure 2).

### 3.5 Autophagy

Autophagy is a highly conserved lysosome-dependent catabolic process in eukaryotic cells that degrades damaged organelles and excess proteins and plays a pivotal role in maintaining intracellular homeostasis. While appropriately regulated autophagy is cytoprotective, both excessive activation and impaired autophagic flux can contribute to cellular injury and death. Experimental evidence indicates that MSC-derived Exos delivering miR-127-3p target KIF3B, inhibit Hedgehog signaling via Gli1 and downregulate the autophagy-related proteins ATG5 and ATG7, thereby restraining excessive autophagy and ameliorating both RIRI and hypoxia–reoxygenation-induced TEC injury (Ji et al., 2025). Conversely, in sepsis-induced AKI, BMSC-derived Exos activate the AMPK/mTOR pathway and enhance adaptive autophagy, which in turn suppresses inflammatory responses and apoptosis (Jin et al., 2023). Together, these findings highlight the context-dependent role of exosome-mediated regulation of autophagy in renal protection.

## 4 Translational applications of Exos in RIRI

### 4.1 Diagnostic applications

Exos are extracellular vesicles released by living cells that partially mirror the physiological and pathological state of their cells of origin. Under physiological conditions, circulating serum Exos do not readily traverse the nephron. Therefore, urinary Exos originate predominantly from renal epithelial cells and are particularly relevant for the early detection of RIRI. Bioactive macromolecules packaged within urinary Exos are therefore promising candidate biomarkers for AKI.

Studies have shown that both the repertoire and abundance of exosomal miRNAs in the plasma or serum of patients with renal cell carcinoma differ significantly from those in healthy individuals, suggesting that dynamic profiling of exosome-derived miRNAs may enable non-invasive monitoring of kidney disease progression (Xiao et al., 2020). For biomarker development, several urinary exosomal microRNAs show early changes in experimental RIRI and, in some settings, in human cohorts linked to ischemic injury. After unilateral RIRI, urinary exosomal miR-150-5p increases, and subsequent work suggests transfer to fibroblasts with pro-fibrotic effects, supporting its candidacy as an early marker (Zhou et al., 2021). In rats, urinary exosomal miR-182 rises within 12 h of ischemia and its temporal pattern tracks histopathology and kidney function measures (Du and Ning, 2021). Urinary exosomal miR-423-5p increases early in RIRI models and has been reported in a cohort of kidney transplant recipients with delayed graft function, where it associates with microvascular rarefaction and declining kidney function (Migneault et al., 2025). Urinary exosomal miR-374b-5p also increases in RIRI models, appears to transfer from injured tubular epithelial cells to macrophages, and promotes M1 polarization. Inhibition of miR-374b-5p attenuates injury, and its early rise supports biomarker potential (Ding et al., 2020). Urinary exosomal miR-125b is similarly increased in rat RIRI models and has been proposed as a candidate marker for early renal IRI (Güçlü et al., 2017). Collectively, these data support urinary exosomal miRNAs, including miR-182, miR-150-5p and miR-423-5p, as candidates for very early RIRI detection, while highlighting the need for harmonized sampling, normalization, and prospective validation in well-defined clinical RIRI settings.

These miRNAs participate in the regulation of glucose metabolism, fibrogenic signaling and apoptotic pathways, underscoring their potential as non-invasive diagnostic and prognostic biomarkers (Resaz et al., 2021). In patients with hypertension and renal injury, plasma Exos are markedly enriched in miR-21-5p, termed the RedoxifibromiR, which simultaneously modulates oxidative stress and fibrotic remodeling. Exosomal miR-21-5p levels correlate closely with renal injury markers such as urinary albumin excretion and show promising early diagnostic performance (Martinez-Arroyo et al., 2025).

More recently, innovative biosensors exploiting split G-quadruplex structures and aggregation-induced emission (AIE) have been developed to detect miRNA signatures enriched in urinary Exos, enabling label-free and ultrasensitive identification of AKI-related miRNAs (Ma et al., 2024). Collectively, these advances highlight the considerable promise of Exos as non-invasive tools for the early diagnosis of AKI and RIRI.

### 4.2 Therapeutic applications

RIRI is a major cause of AKI, and its progression is typically accompanied by gradual loss of renal function that often culminates in chronic kidney disease. Current therapeutic options are largely limited to hemodialysis and renal transplantation, and in the context of donor organ shortage long-term outcomes remain suboptimal for many patients. These limitations underscore the urgent need for novel therapies and position exosome-based interventions as particularly attractive candidates. Exos exhibit intrinsic homing properties that enable selective targeting of injured tissues or specific cell populations and thereby facilitate repair and regeneration. Compared with direct cell therapy, Exos do not form cellular emboli, elicit weaker alloimmune responses and better preserve hemodynamic stability, thereby offering a safer therapeutic profile. Exos are also highly engineerable; their cargo composition and release profiles can be tailored to achieve precise delivery of therapeutic molecules. Moreover, they are relatively stable, amenable to cryopreservation and retain bioactivity after storage, characteristics that favor large-scale production, storage and clinical translation.

#### 4.2.1 Potential detrimental effects

As outlined above, Exos mitigate RIRI through multiple mechanisms, including suppression of oxidative stress and inflammation, inhibition of apoptosis and epithelial–mesenchymal transition and attenuation of AKI-to-CKD progression. However, Exos are also rich in non-coding RNAs, some of which may exert pro-inflammatory, pro-injury, and pro-fibrotic effects. For example, Exos released by injured TECs can act on neighbouring healthy nephron segments and promote the development of renal fibrosis. In calcium oxalate-induced renal injury models, Exos derived from normal HK-2 cells exert protective effects, whereas Exos released from HK-2 cells stimulated by calcium oxalate crystals exacerbate oxidative stress, drive macrophage polarization toward a pro-inflammatory M1 phenotype and aggravate inflammation, renal injury and stone formation (Yang Y. et al., 2025). In a separate calcium oxalate crystal–induced injury model, TEC–derived Exos activated transcription of miR-93-3p via the CREB1/CRTC2 axis. Following uptake by macrophages, miR-93-3p suppresses NFAT5, activates the NIK/NF-κB2 pathway, promotes M1 macrophage polarization and macrophage extracellular trap (MET) formation and further exacerbates renal inflammation and damage (Sun et al., 2025). Li et al. suggested that TEC–derived Exos promote macrophage polarization toward a pro-inflammatory M1 phenotype by activating the miR-106b-5p/ATL3 signaling pathway (Li et al., 2023).

Collectively, these findings underscore the potential detrimental roles of specific exosomal cargo and suggest that targeted antagonism of pathogenic miRNAs and blockade of downstream signaling pathways may offer mechanism-based strategies to curb the progression of RIRI at its source.

#### 4.2.2 Engineering approaches

Beyond serving as passive carriers, Exos can be engineered to enhance or redirect their therapeutic functions. Xie et al. reported that BMSC-Exos engineered to overexpress indoleamine 2,3-dioxygenase (IDO) modulate macrophage polarization and thereby attenuate the progression of RIRI (Xie et al., 2022). In another approach, TEC–derived Exos were engineered to overexpress CD26, resulting in increased PCNA expression, reduced p53/p21 levels, and enhanced tubular cell proliferation. Concomitant downregulation of the CXCR4/SDF1 axis reduced macrophage and neutrophil infiltration, thereby dampening inflammation and mitigating renal IRI (Du et al., 2021). Exos derived from ADSCs engineered to overexpress HOXB3OS displayed superior renoprotective effects compared with unmodified Exos, leading to more pronounced improvements in renal function and structural integrity in both *in vitro* and *in vivo* models (Wang et al., 2025).

A B7-H1-high subpopulation of hHCMSCs was also identified, and Exos derived from these cells (B7-H1^high-Exos) exhibited enhanced reparative capacity in renal IRI models by downregulating complement C3 and NF-κB signaling, thereby reducing inflammation and oxidative stress (He et al., 2025). These findings support “pre-engineering” donor cells as a feasible strategy to optimize the therapeutic profile of their Exos. In addition, pretreatment of Umbilical cord-MSCs (UC-MSCs) with the herbal compound puerarin was shown to suppress lncRNA NEAT1, thereby relieving its sponging of miR-342-3p and enriching miR-342-3p in secreted Exos. These Exos effectively attenuated pyroptosis in TECs, further illustrating the potential of pharmacological preconditioning to engineer exosomal cargo (Yang J. et al., 2025). Overall, engineered Exos represent versatile therapeutic carriers with considerable potential for future clinical application.

#### 4.2.3 Delivery systems and carrier platforms

Systemically administered extracellular vesicles are rapidly cleared by the mononuclear phagocyte system, with an *in vivo* half-life of only a few minutes, which necessitates the development of targeted and sustained-release delivery systems for exosome-based therapies. Hydrogels have emerged as attractive biomaterials because they form highly hydrated three-dimensional polymer networks, typically generated from hydrophilic or amphiphilic polymers through chemical or physical crosslinking. The hydrogel matrix shields cells and labile biomolecules such as proteins, peptides and nucleic acids from harsh environmental conditions and thereby enhances their stability and bioavailability. Han et al. showed that MSCs cultured in 5% GelMA hydrogels produce substantially more Exos than in conventional two-dimensional culture, and that these three-dimensional Exos are smaller, enriched in reparative cargo, and more readily internalized by target cells (Han et al., 2023). Wang et al. used a two-component supramolecular hydrogel composed of glutamine and benzaldehyde derivatives as a delivery matrix for endothelial cell-derived Exos. This hydrogel–Exos composite reduced pro-inflammatory Ly6C^high^ monocytes, macrophages and neutrophils, decreased microvascular thrombosis in infarcted myocardium, improved endothelial barrier integrity and increased microvessel density within the injured region (Wang J. et al., 2023).

Beyond hydrogels, Tang et al. engineered a red blood cell-derived exosomal small interfering RNA delivery system (REVLTH-siP65/siSnai1) targeting the transcription factors p65 and Snai1, which are upregulated in ischemic kidneys. By decorating Exos with the LTHVVWL peptide, which binds kidney injury molecule 1 (Kim-1) with high affinity, they achieved kidney-targeted delivery of siP65 and siSnai1, reduced p65 and Snai1 expression and enhanced renal repair (Tang et al., 2021). Building on the anti-inflammatory and pro-angiogenic properties of MSC-Exos, Hu et al. developed a bioreactive exosome-eluting stent (EES) using MSC-Exos as a biological coating. This platform maintained vessel patency while delivering MSC-Exos locally to ischemic renal tissue, promoted endothelial proliferation and angiogenesis and reduced inflammation and smooth muscle cell migration (Hu et al., 2021). Compared with bare metal stents, EES yielded smaller neointimal areas, and compared with drug-eluting stents it achieved superior strut coverage. Another strategy involves encapsulating MSC-Exos within hyaluronic acid-based dissolvable microneedle patches. These Exos-loaded patches enable local sustained release of Exos and outperform free Exos in modulating apoptosis-related genes such as BAX/BCL-2 and miR-34a, reducing oxidative stress and improving renal function (Taghavi et al., 2025).

In targeted delivery paradigms, Exos can function both as therapeutic cargo and as natural carrier vesicles. Although a variety of synthetic nanocarriers, such as liposomes, dendrimers and calcium phosphate nanoparticles, have been developed, they often exhibit immunogenicity, cytotoxicity and rapid clearance. By contrast, Exos naturally display membrane proteins and lipids that facilitate targeted interaction with recipient cells and enable more precise delivery of therapeutic cargo. For example, in studies using fibroblastic reticular cell-derived Exos, LTH peptide modification was used to enhance selective targeting to injured renal tubules (Li et al., 2024). Using EXPLOR optogenetic engineering technology, Exos have been loaded with a super-repressor form of IκBα (srIκB) and systemically administered to mice, leading to inhibition of NF-κB signaling, amelioration of RIRI, improved renal function and reduced inflammatory cytokines, apoptotic markers and immune cell infiltration (Kim et al., 2021). In another study, MSCs were transfected with a let-7i-5p antagonist, and the resulting MSC-Exos delivered this antagonist to TECs and thereby suppressed fibrotic processes (Jin et al., 2021).

**FIGURE 4:**
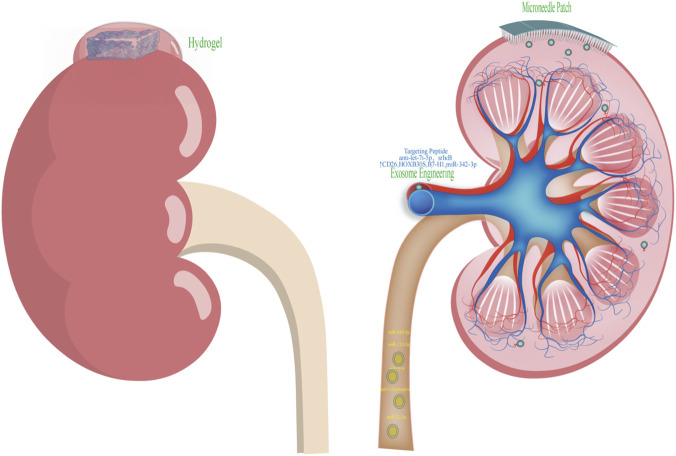
Schematic illustration of Exos engineering and delivery strategies: surface modification via targeting peptides, encapsulation within hydrogels and microneedle patches for therapeutic applications, and the diagnostic potential of urinary exosomal miRNAs for early-stage detection.

Taken together, the unique attributes of Exos as natural targetable drug carriers confer considerable potential for their application in RIRI. Specifically, RIRI is a principal driver of AKI and involves intertwined mechanisms of oxidative stress, inflammatory cascades, mitochondrial dysfunction and programmed cell death. Exos can be harnessed to deliver therapeutic payloads such as antioxidants or anti-apoptotic agents, and, analogous to rabies virus glycoprotein (RVG) peptide-based brain-targeting strategies, kidney-specific ligands can be displayed on their surface through genetic or chemical engineering to enhance accumulation in injured renal tissue. Nonetheless, kidney-targeted Exos carrier systems remain relatively underexplored. Such platforms may overcome key limitations of conventional nanomedicines and open new avenues for the treatment of AKI and CKD, underscoring the need for further preclinical optimization and clinical translation (Figure 4).

### 4.3 Research limitations and translational barriers

Exos from distinct cellular sources can modulate oxidative stress, inflammation, and repair via RNAs and proteins, but mechanisms are not necessarily interchangeable across sources (Zhang et al., 2025). Stem cell-derived Exos offer broad immunomodulatory capacity but can vary batch-to-batch. Tubular epithelial cell-derived Exos may better reflect the target tissue yet remain sensitive to injury-associated microenvironments. Cargo composition is tightly coupled to the state of the parent cell, which likely contributes to divergent reports of antioxidant and anti-inflammatory effects. A further limitation is the paucity of direct comparative studies that evaluate Exos from different sources within the same experimental platform and injury model (Cao et al., 2025). Differences in injury paradigms, dosing, delivery routes, and endpoints also restrict cross-study comparisons (Himanshu et al., 2025). Context dependence adds another layer of complexity, because pathways such as autophagy can be protective or harmful depending on timing and tissue state (Romero et al., 2023). A standardized comparative framework is therefore needed to define when source-specific Exos are most effective, particularly in pathways linked to programmed cell death, proliferation, and autophagy.

Key translational barriers remain underdeveloped in the literature and deserve explicit consideration. Standardization of isolation, characterization, and reporting is essential for reproducibility, and community frameworks such as the ISEV MISEV guidance provide a baseline for study design and disclosure, including blood-specific reporting through MIBlood-EV. However, a recent survey regarding the MIBlood-EV reporting framework—introduced in 2023 specifically for blood-derived EVs—highlighted a significant gap in current research practices: although 95% of investigators are aware of the broader MISEV guidelines, only 65% actually implement them in their publications, and 40% remain unaware of the MIBlood-EV specifications (Nieuwland et al., 2025). Consistent with these findings, systematic evaluations reveal that a majority of studies fail to strictly adhere to the MISEV guidelines, exhibiting notable deficiencies particularly in the characterization of cellular sources and the reporting of bioactive cargo. Consequently, there is an urgent need to enforce methodological standardization to enhance study reproducibility and ensure therapeutic safety (Rahman et al., 2025). Translation also requires quantitative understanding of biodistribution and pharmacokinetics, including tissue uptake, clearance, dose-exposure relationships, and route-dependent delivery to the injured kidney. Safety should be assessed systematically, not only for the vesicles themselves but also for process-derived risks introduced during isolation and purification, including residual cell debris, endotoxin, and other impurities, as well as unintended immune activation and off-target effects (Wen et al., 2025). These issues are amplified for engineered Exos, where surface modification and cargo loading introduce additional manufacturing variables and complicate comparability across batches. Progress toward clinical application will likely depend on harmonized quality controls, validated potency assays, and a clearer regulatory pathway that links product attributes to mechanism and clinical risk (Zhang et al., 2023; Rahbarghazi et al., 2019). To date, no registered or ongoing clinical trial has specifically validated the intervention effect and safety of Exos in human RIRI, primarily due to the field generally lacking unified standards for isolation and purification, and drug loading efficiency needing improvement (Liu C. et al., 2023). These factors pose technical challenges for translating exosome-based RIRI therapies into human application. Furthermore, for this specific indication, there remains a lack of systematic, standardized non-clinical safety data, which has slowed the pace of advancing into human clinical trials. While animal model-based RIRI studies have provided theoretical rationale and established preliminary evidence of efficacy for exosome therapy, its clinical translation remains in the very early exploratory stages compared to fields such as dermatology and oncology. To advance the application of Exos in treating human RIRI in the future, the field must draw on experience from other disciplines, establish a comprehensive Exos safety evaluation system, and conduct large-scale, multicenter clinical trials to generate critical long-term safety and efficacy data (Moslehi et al., 2026).

## 5 Summary and perspectives

RIRI, a major precipitating factor of AKI, is characterized by complex pathophysiology and limited effective therapeutic options. In recent years, Exos have emerged as promising tools for both the diagnosis and treatment of RIRI owing to their unique biological properties. As extracellular vesicles enriched in diverse RNAs and proteins, Exos encapsulate miRNAs and proteins such as ATF3 and AQP1 that reflect early injury in renal TECs. Urine-derived Exos, in particular, offer a non-invasive and highly sensitive window into renal pathology and provide a novel avenue for the early diagnosis of RIRI.

A growing body of preclinical evidence indicates that Exos modulate RIRI through multiple mechanisms, including attenuation of oxidative stress and inflammation, the inhibition of apoptosis and epithelial–mesenchymal transition, and promotion of tissue repair and regeneration. In addition, Exos can be enhanced by engineering strategies such as gene modification exemplified by IDO overexpression, display of targeting peptides such as the KSP peptide and drug loading *via* ultrasound or electroporation, achieving more precise tissue targeting and greater therapeutic efficacy. Advanced delivery platforms, including hydrogel-based systems, can further extend exosomal half-life *in vivo* and augment their local accumulation at sites of injury.

Nevertheless, Exos can also act as double-edged swords. Under specific conditions, such as hypoxia-induced enrichment of pro-inflammatory miR-23a, Exos may exacerbate tissue damage, highlighting both potential risks and therapeutic opportunities for selectively neutralizing pathogenic exosomal cargo or their downstream signaling. Despite these advances, important hurdles still impede clinical translation, including standardization of Exos isolation and purification, potential interspecies immune incompatibility and the need for rigorous assessment of exosomal immunogenicity, tumorigenic risk and *in vivo* pharmacokinetics and metabolism. These challenges, however, do not negate the considerable promise of exosome-based approaches. Exos may ultimately serve as integrated diagnostic and therapeutic platforms, informing clinical decision-making and enabling mechanism-based, individualized treatment strategies tailored to distinct stages and phenotypes of RIRI.

In summary, Exos, as natural nanoscale diagnostic and therapeutic platforms, are driving a shift in the RIRI field from purely mechanistic insights toward translational and clinically oriented applications, although important scientific and translational challenges remain. Looking forward, advances in bioengineering and cross-disciplinary collaboration may further develop exosome-based strategies for RIRI and other kidney diseases and support a transition in AKI management from symptomatic supportive care toward mechanism-based, targeted renal repair. In this review, we summarize recent advances in Exos research in RIRI, delineate the diagnostic and therapeutic potential of Exos from diverse cellular origins and highlight how bioengineering strategies can further refine and prolong their renoprotective effects. Collectively, ongoing and future studies on Exos are likely to be important for advancing the prevention, early detection and precise treatment of RIRI.
